# An Elementary Treatise on Human Anatomy

**Published:** 1861-01

**Authors:** 


An Elementary Treatise on Human Anatomy. By Joseph Leidy, M. D., Prof. of Anatomy in the University of Pennsylvania, &c., &c., with 392 illustrations. Philadelphia, J. B. Lippincott & Co.—1861.

This a good sized octavo volume of 663 pages, published in superb style. It adds another to the many elementary treatise or text-books, on human anatomy, that have been issued from the Medical Press during the last six or eight years. From a hasty glance at the contents of the book, together with the well known ability of the author, we should think it admirably adapted for use as a text-book for the student. Its arrangement is simple, its style direct and plain, and its illustrations excellant. For sale by S. C. Griggs & Co., Chicago.

